# Geographical and socioeconomic inequalities in the utilization of maternal healthcare services in Nigeria: 2003–2017

**DOI:** 10.1186/s12913-020-05700-w

**Published:** 2020-09-10

**Authors:** Chijioke Okoli, Mohammad Hajizadeh, Mohammad Mafizur Rahman, Rasheda Khanam

**Affiliations:** 1 School of Commerce, and Centre for Health Research, University of Southern Queensland, Toowoomba, QLD 4350 Australia; 2 Department of Health Administration and Management, Faculty of Health Sciences Technology, College of Medicine, University of Nigeria, Enugu Campus, Enugu, Enugu State Nigeria; 3 School of Health Administration, Dalhousie University, Halifax, Canada

**Keywords:** Geographical inequalities; socioeconomic inequalities, Maternal healthcare, Nigeria

## Abstract

**Background:**

Maternal mortality has remained a challenge in many low-income countries, especially in Africa and in Nigeria in particular. This study examines the geographical and socioeconomic inequalities in maternal healthcare utilization in Nigeria over the period between 2003 and 2017.

**Methods:**

The study used four rounds of Nigeria Demographic Health Surveys (DHS, 2003, 2008, 2013, and 2018) for women aged 15–49 years old. The rate ratios and differences (RR and RD) were used to measure differences between urban and rural areas in terms of the utilization of the three maternal healthcare services including antenatal care (ANC), facility-based delivery (FBD), and skilled-birth attendance (SBA). The Theil index (T), between-group variance (BGV) were used to measure relative and absolute inequalities in the utilization of maternal healthcare across the six geopolitical zones in Nigeria. The relative and absolute concentration index (RC and AC) were used to measure education-and wealth-related inequalities in the utilization of maternal healthcare services.

**Results:**

The RD shows that the gap in the utilization of FBD between urban and rural areas significantly increased by 0.3% per year over the study period. The Theil index suggests a decline in relative inequalities in ANC and FBD across the six geopolitical zones by 7, and 1.8% per year, respectively. The BGV results do not suggest any changes in absolute inequalities in ANC, FBD, and SBA utilization across the geopolitical zones over time. The results of the RC and the AC suggest a persistently higher concentration of maternal healthcare use among well-educated and wealthier mothers in Nigeria over the study period.

**Conclusion:**

We found that the utilization of maternal healthcare is lower among poorer and less-educated women, as well as those living in rural areas and North West and North East geopolitical zones. Thus, the focus should be on implementing strategies that increase the uptake of maternal healthcare services among these groups.

## Background

Despite continual efforts to reduce maternal mortality burden globally, it has remained an ongoing tragedy in many low-income countries, especially in Africa [1–4], which has the highest rate of maternal deaths in the world and sub-Saharan Africa as a primary contributor has a maternal death of 1 in every 16 pregnant women compared with 1 in 2800 in the developed countries [5]. This substantial difference is one of the largest inequalities of any public health statistics [6].

Social inequalities that prevail in the health sector especially between the poor and the rich continue to be a cause for concern, particularly in the developing worlds [7]. These inequalities are manifested in health outcomes as studies in developing countries show that maternal health service utilization is higher among wealthier women than their poorer counterparts [7–9], mostly residing in rural areas [10]. Living in rural areas in developing countries mean residing in deprived communities in terms of social amenities and infrastructure [8].

The rural-urban place of residence accounts for differences in the use of health services, especially as this relates to the level of maternal education and socioeconomic status [5, 8]. Studies show a positive association between education level and the use of antenatal care (ANC), delivery in health facilities (FBD), and skilled birth assistance (SBA) [8]. Of equal importance, is socioeconomic status, which influences the use of health services as the wealthier urban women access healthcare more compared to their poorer rural counterparts.

In Nigeria, there has been some decline in maternal mortality from 576 per 100,000 live births in 2013 to 512 per 100,000 live births in 2018 [11]. The pace of reduction and geographical inequalities in the distribution remains a huge concern. There are inequities in maternal mortality rate across the six geopolitical zones in Nigeria with North-East and North-West zones of the country having almost 10 and 6 times, respectively, higher mortality rates than that of the South-West zone of the country [11]. Women from northern Nigeria, especially in rural areas, are at higher risk of maternal death compared to those from the southern part of the country [11]. Lower access to health care services is most common in the Northern zones of the country, particularly in rural areas, among low socioeconomic status (SES) individuals [11]. This is due to distance to facility, limited means of transportation, poor staffing of the health facilities, poor attitude/unprofessional conduct of healthcare providers, and lower levels of education [12–15].

To date, most studies in Nigeria focus mainly on socioeconomic inequalities in maternal mortality rates [5, 16]. There is a paucity of studies in the literature assessing geographical and socioeconomic inequalities in maternal healthcare use in Nigeria. Using information collected from the four cycles of the Nigeria Demographic Health Surveys (DHS, 2003, 2008, 2013 and 2018), this study examines trends in the geographical and socioeconomic inequalities in maternal healthcare services utilization over the period between 2003 and 2017. The results of this study will provide useful information for policymakers to address geopolitical socioeconomic inequalities in maternal healthcare services that determine health outcomes in the country.

## Methods

### Study setting

The study setting is in Nigeria, with an estimated population of 198 million as of 2018 [11]. The country comprises 36 states and a Federal Capital Territory, Abuja. The country is divided into six geopolitical zones for administrative and political purposes (North-Central, North-East, North-West, South-East, South-West, and South-South). These geopolitical zones comprise states with a similar culture, ethnic groups, and common history [1, 11].

The country has a three-tiered health system; primary, secondary, and tertiary based on the three tiers of government – local, state, and federal. More health services providers are located in the southern than in the northern states of Nigeria, [17], owing to widespread poverty in the North than in the South [18], but there are some other significant issues: for example, fewer than 20% of healthcare facilities in the country offer emergency obstetric care [11]. In terms of levels of socioeconomic development, wide differences exist between the northern and the southern parts of the country and across the geopolitical zones [10]. Approximately 62% of Nigerians live below the poverty line [10], with northern geopolitical zones having the highest poverty rates in the country [19].

### Data

Of the available five rounds of the Nigeria demographic and health survey (1990, 2003, 2008, 2013 and 2018), this study used the latest four. The 1990 DHS was not included because the survey was limited to four (North-East, North-West, South-East, and South-West) of the six geopolitical zones of Nigeria. The Nigerian DHS is part of the DHS program designed to collect nationally representative information using three types of structured questionnaires: household questionnaire, women’s questionnaire, and, men’s questionnaire [10, 20]. The survey used a three-stage cluster sampling design and covered all the six geopolitical zones of the country. The sampling frame was based on the list of enumeration areas prepared for the 1991 and 2006 Population Census of the Federal Republic of Nigeria. Details of the survey have been provided elsewhere [21]. This study utilizes the information collected through the women’s questionnaire on issues related to maternal and child health, fertility, and family planning for women aged 15–49.

### Variables

#### Outcome measures

The outcome variables of the study are three key aspects of maternal healthcare ANC, FBD, and SBA. Based on the recommendations of the World Health Organization (WHO), an ANC visit is defined as a pregnant woman having at least four antenatal assessments by or under the supervision of a skilled attendant [22]. Although the 2016 WHO guideline stipulates eight ANC visits [23], we used the old guidelines as data came mostly from the period with four ANC visits.

The FBD is defined as giving birth at a permanent health-facility such as primary health centers, hospitals, or a private clinic. The SBA is defined as delivery assisted by an accredited health professional such as a doctor, nurse, midwife, or an auxiliary nurse/midwife [20, 21].

#### Socioeconomic variables

Maternal education and household wealth index (WI) were used as socioeconomic variables in the study. The WI was measured using household asset ownership, household characteristics, household source of drinking water, and household sanitary facilities as contained in DHS datasets [21, 24]. The WI is generally used as an indicator for household SES when income or expenditure data is unavailable [25]. The WI is constructed using principal components analysis (PCA) technique that assigns a score to each household based on selected household assets. The first principal component of a set of variables captures the largest amount of information that is common to all the variables [26, 27]. The mother’s education level (in years) was used as another measure of SES in the study [20].

### Statistical analysis

Our statistical analysis involved measuring geographic, education, and wealth-related inequalities. We calculated geographic inequalities in the utilization of maternal healthcare services (ANC, FBD, and SBA) between urban and rural areas and across the six geopolitical zones of Nigeria. Education and wealth-related inequalities in access to maternal healthcare were also estimated for the study period. The chi-square test was set at 0.05% level of significance. Weights were applied to ensure the representativeness of the actual population.

#### Measuring inequalities between urban and rural and across geopolitical zones

Absolute and relative inequalities between urban and rural areas were calculated using rate ratio (RR) and rate difference (RD). The Theil index (T) was employed to estimate relative inequalities in maternal healthcare utilization between the six geopolitical zones [20, 28]. The T can be estimated as follows:

1 $$ T={\sum}_{i=1}^i{GZ}_{ih}\left[\ln \left(\frac{GZ_{ih}\ }{GZ_{ip}\ }\right)\right], $$

where *GZ*_*ih*_ is the geopolitical zone’s share of the population’s health and *GZ*_*ip*_ is the *i*_th_ zone’s population share. The T ranges from zero, indicating an equal distribution, while a higher value suggests a more unequal distribution. Moreover, the between-group variance (BGV) was used to summarize absolute inequality across the geopolitical zones [20, 28]. The BGV was calculated as:

2 $$ BGV={\sum}_{i=1}^i{GZP}_i{\left({GZH}_i-\mu \right)}^2 $$

Where *GZP*_*i*_ is geopolitical zone ’s population size (i.e., number of women who gave birth in each year), *GZH*_*i*_ is geopolitical zone *i*’s average health outcome, *μ* is the average health outcome across all the geopolitical zones.

#### Measuring socioeconomic inequalities

The concentration index (C index) approach was used to calculate socioeconomic related inequalities in the utilization of maternal healthcare services. The index is a widely used measure of socio-economic health inequalities as it fulfills three qualities for a valid socioeconomic inequality index. The index should: a) reflect the health inequalities that arise from the socioeconomic characteristics; b) be representative of the whole population; and c) be sensitive to the subpopulation group sizes [29, 30]. The C index quantifies the extent of socioeconomic inequality in health, which is useful in tracing inequalities over time across different groups [29].

The relative concentration index (RC) is based on the relative concentration curve which graphs the cumulative share of maternal healthcare use (e.g., ANC), on its y-axis, against the cumulative share of the population, ranked in ascending order of an SES indicator (e.g. the WI) on its x-axis. The RC is calculated as twice the area between the relative concentration curve and the perfect equality line. The RC is negative (positive) if the concentration curve lies above (below) the line of equality, indicating that the utilization of maternal healthcare service is concentrated among poorer (richer) women [31, 32]. The RC ranges from − 1 to 1, with a value of zero signifying “perfect equality” [29]. The convenient regression method can be used to compute the RC index as follows [32]:

3 $$ 2{\sigma}_r^2\left(\frac{y_i}{\mu}\right)=\alpha +\varphi {r}_i+{\varepsilon}_i, $$

where *y*_*i*_ is the healthcare variable of interest (e.g. ANC) for women *i*, *μ* is the mean of the healthcare utilization variable for the whole sample, *r*_*i*_ *= i/N*, is the fractional rank of individual *i* in the distribution from the lowest SES woman (*i* = 1) to the highest SES woman (*i* = *N)*, and ${\sigma}_r^2$ is the variance of fractional rank. The RC is calculated as the ordinary least squares (OLS) estimate of *φ* [33].

Since our outcome variable of interest is binary, the minimum and maximum values of the RC are not − 1 and + 1, thus, the RC was normalized by multiplying the estimated index by 1/1-μ, where μ indicates the mean of outcome variable of interest [34, 35]. The generalized concentration index (*RC* × *μ*) can be used to calculate absolute socioeconomic inequality in healthcare utilization [31]. Since the generalized concentration index does not satisfy this condition, the Erreygers modified the generalized/absolute concentration index (hereafter the =*RC* × 4*μ*) [34, 36] was used to calculate absolute socioeconomic inequality in healthcare utilization. The AC ranges from − 1 to + 1, with zero suggesting perfect equality [34]. All analyses were weighted to account for individual survey sample designs. All analyses were conducted using version 13 of the STATA software package (Stata Corp, College Station, Tex).

## Results

### Descriptive statistics

Table 1 shows maternal healthcare utilization by the sample characteristics. Of the three age groups, women aged 25–34 years, on average use more maternal ANC, FBD, and SBA over the four-year survey periods. Those with secondary education levels on average utilizes more maternal healthcare services than those with no formal education, or education at primary or tertiary education levels. Expectedly, married women use more ANC, FBD, and SBA than the never married and others (divorced, living together, not living together, and widowed). In the same vein, those employed or working on average use more maternal healthcare than their employed counterparts.

**Table 1:** Maternal healthcare utilization in Nigeria by mother’s characteristics and geographic regions (2003–2018)

	2003			2008			2013			2018		
	ANC	FBD	SBA	ANC	FBD	SBA	ANC	FBD	SBA	ANC	FBD	SBA
**Age of women**												
15–24	458(25.7)	436(22.8)	483(23.2)	1712(21.4)	1783(19.3)	1974(19.6)	2339(22.7)	2292(20.9)	2427(20.6)	2708(22.3)	2570(19.6)	2785(19.3)
25–34	852(47.9)	1052(55.1)	1120(53.8)	4167(52.1)	5203(56.2)	5645(56.0)	5116(49.7)	5881(53.7)	6360(54.1)	6047(49.8)	7138(53.4)	7896(54.7)
35–49	469(26.4)	421(22.1)	481(23.1)	2123(26.5)	2264(24.5)	2457(24.4)	2838(27.6)	2778(25.4)	2978(25.3)	3394(27.9)	3421(26.0)	3743(26.0)
**Education level**												
No formal education	477(27.0)	312(16.4)	390(18.7)	1820(22.8)	1229(13.3)	1452(14.4)	2660(25.8)	1702(15.4)	17,794(15.3)	3346(27.5)	2171(16.5)	2282(15.8)
Primary	522(29.5)	564(30.0)	618(29.6)	2178(27.2)	2405(26.0)	2655(26.4)	2344(22.8)	2454(22.4)	2639(22.4)	1994(16.4)	2019(15.4)	2289(15.9)
Secondary	648(36.6)	853(44.7)	893(42.9)	3126(39.1)	4345(47.0)	4660(46.3)	4164(40.5)	5196(47.4)	5686(48.3)	5125(42.2)	6543(49.8)	7311(50.7)
Tertiary	123(7.0)	180(9.4)	183(8.8)	878(11.0)	1270(13.7)	1309(13.0)	1125(10.9)	1599(14.6)	1647(14.0)	1683(13.9)	2395(18.3)	2543(17.6)
**Marital status**												
Never married	63(3.6)	75(3.9)	75(3.6)	198(2.5)	172(1.9)	193(1.9)	299(2.9)	241(2.2)	262(2.2)	290(2.4)	299(2.3)	337(2.3)
Married	1553(87.7)	1663(87.1)	1827(87.7)	7364(92.0)	8614(93.1)	9342(92.7)	9276(90.1)	9988(91.2)	10,702(91.0)	10,956(90.2)	11,872(90.4)	12,998(90.1)
Others	154(8.7)	171(9.0)	181(8.7)	440(5.5)	464(5.0)	540(5.4)	718(7.0)	723(6.6)	802(6.8)	902(7.4)	958(7.3)	1089(7.6)
**Work status**												
Not working	505(28.6)	550(28.9)	606(29.2)	2092(26.3)	2191(23.8)	2292(25.5)	2542(24.8)	2533(23.3)	2756(23.6)	3081(25.4)	3022(23.0)	3322(23.0)
working	1262(71.4)	1352(71.1)	1470(70.8)	5870(73.7)	7009(76.2)	6688(74.5)	7705(75.2)	8356(76.7)	8944(76.4)	9067(74.6)	10,107(77.0)	11,103(77.0)
**Religion**												
Christian	933(52.7)	1227(64.3)	1301(62.5)	4679(58.8)	6308(68.6)	6818(68.0)	5409(53.0)	6921(63.9)	7447(64.0)	6013(49.7)	7878(60.3)	8734(60.8)
Muslim	817(46.2)	663(34.8)	764(36.7)	3183(40.0)	2827(30.7)	3132(31.2)	4726(46.3)	3850(35.5)	4128(35.5)	6055(50.1)	5174(39.6)	5613(39.1)
Other	19(1.1)	18(0.9)	17(0.8)	89(1.1)	67(0.7)	75(0.8)	70(0.7)	63(0.6)	59(0.5)	29(0.2)	24(0.2)	27(0.2)
**Place of residence**												
Urban	774(43.7)	920(48.2)	1003(48.1)	3672(46.0)	4756(51.5)	5087(50.6)	5295(51.9)	6521(60.1)	7140(61.4)	6184(51.1)	7756(59.3)	8562(59.6)
Rural	996(56.3)	988(51.8)	1080(51.9)	4318(54.0)	4477(48.5)	4970(49.4)	4909(48.1)	4313(39.8)	4495(38.6)	5913(48.9)	5320(40.7)	5813(40.4)
**Wealth index**												
poorest	191(10.8)	153(8.0)	155(7.4)	653(8.2)	469(5.1)	531(5.3)	829(8.1)	422(3.9)	406(3.5)	1430(11.8)	868(6.6)	880(6.1)
Poorer	255(12.7)	208(10.9)	233(11.2)	1147(14.4)	916(9.9)	1052(10.5)	1565(15.3)	1212(11.2)	1238(10.6)	2039(16.9)	1616(12.4)	1694(11.8)
Middle	345(19.5)	297(15.5)	315(15.1)	1637(20.5)	1653(17.9)	1837(18.3)	2185(21.4)	2127(19.6)	2312(19.9)	2632(21.8)	2783(21.3)	3151(21.9)
Richer	450(25.5)	482(25.2)	560(26.9)	2078(26.0)	2596(28.1)	2869(28.5)	2620(25.7)	3066(28.3)	3364(28.9)	2882(23.8)	3586(27.4)	4040(28.1)
Richest	559(31.6)	769(40.3)	821(39.4)	2474(31.0)	3600(39.0)	3769(37.5)	3006(29.5)	4007(37.0)	4315(37.1)	3114(25.7)	4223(32.3)	4611(32.1)
**Geopolitical zones**												
North-central	307(17.4)	388(20.3)	423(20.3)	1270(15.9)	1560(16.9)	1614(16.1)	1553(15.2)	1882(17.4)	1938(16.7)	1620(13.4)	2244(17.2)	2352(16.4)
North-east	268(15.2)	239(12.5)	281(13.5)	932(11.7)	596(6.5)	718(7.1)	1305(12.8)	1045(9.7)	1074(9.2)	1690(14.0)	1562(12.0)	1547(10.8)
North-west	367(20.7)	215(11.2)	257(12.3)	1154(14.5)	679(7.4)	786(7.8)	2214(21.7)	1322(12.2)	1430(12.3)	3213(26.6)	1936(14.8)	2301(16.0)
south-east	153(8.6)	276(14.5)	289(13.9)	985(12.3)	1789(19.4)	1924(19.1)	1381(13.5)	2104(19.4)	2237(19.2)	1752(14.5)	2758(21.1)	2898(20.2)
South-south	354(20.0)	402(21.0)	425(20.4)	1250(15.7)	1545(16.7)	1780(17.7)	1216(11.9)	1333(12.3)	1670(12.6)	1299(10.7)	1406(10.8)	1807(12.6)
South-west	321(18.1)	390(20.4)	409(19.6)	2398(30.0)	3064(33.2)	3235(32.2)	2537(24.9)	3147(29.1)	3487(30.0)	2524(20.9)	3170(24.2)	3470(24.1)

*ANC* Antenatal care, *FBD* Facility based delivery, *SBA* Skilled birth attendance

The results show Christians utilize more maternal healthcare services compared to Muslims and other religions. For the place of residence, urban residents used more maternal care services than rural residents. However, the wealth index shows a positive relationship in maternal healthcare utilization. Of the six geopolitical zones, the average utilization of maternal care use was higher in South-West followed by North-Central zones while it was lower in North-West and North-East zones.

Table 2 reports the survey year, sample size, and average utilization rates for ANC, FBD, and SBA for the total population (the six geopolitical zones) and urban and rural areas for each year within the survey periods. The total measures of maternal healthcare utilization increased for ANC, FBD, and SBA among women who gave birth between 1998 and 2017. The results show that only 58, 32 and 14% of women who gave birth in 1998 used ANC, FBD, and SBA respectively, while these figures increased to 58, 42, and 45%, respectively in 2017. The utilization of maternal healthcare services also increased in urban and rural areas in Nigeria.

**Table 2:** Survey year, sample size, and maternal care use (mean) in Nigeria, 2003–2018

Survey year	Survey year	Sample size	ANC			FBD			SBA		
			Total	Urban	Rural	Total	Urban	Rural	Total	Urban	Rural
2003	1998	1120	0.58	0.76	0.49	0.32	0.58	0.19	0.14	0.30	0.08
	1999	1194	0.52	0.67	0.46	0.28	0.51	0.20	0.31	0.55	0.23
	2000	1246	0.51	0.78	0.41	0.36	0.57	0.27	0.38	0.60	0.30
	2001	1129	0.50	0.73	0.39	0.36	0.54	0.26	0.37	0.58	0.27
	2002	1368	0.47	0.72	0.37	0.34	0.52	0.26	0.36	0.58	0.27
2008	2003	4933	0.66	0.83	0.57	0.44	0.69	0.33	0.11	0.18	0.08
	2004	5701	0.57	0.78	0.45	0.35	0.62	0.24	0.36	0.64	0.25
	2005	5827	0.51	0.82	0.38	0.33	0.59	0.23	0.36	0.64	0.25
	2006	5640	0.48	0.76	0.37	0.34	0.61	0.23	0.37	0.65	0.26
	2007	6032	0.49	0.77	0.37	0.34	0.62	0.23	0.37	0.66	0.26
2013	2008	6561	0.57	0.82	0.39	0.39	0.64	0.23	0.23	0.42	0.13
	2009	6094	0.55	0.81	0.38	0.33	0.60	0.19	0.36	0.65	0.20
	2010	6356	0.51	0.76	0.37	0.35	0.60	0.21	0.37	0.65	0.22
	2011	6054	0.55	0.79	0.42	0.38	0.64	0.23	0.40	0.70	0.24
	2012	7167	0.52	0.77	0.39	0.37	0.62	0.23	0.39	0.68	0.24
2018	2013	6756	0.71	0.80	0.58	0.50	0.70	0.33	0.07	0.12	0.04
	2014	7068	0.65	0.82	0.51	0.38	0.60	0.24	0.41	0.65	0.26
	2015	6997	0.61	0.78	0.47	0.38	0.62	0.24	0.41	0.66	0.26
	2016	6612	0.56	0.75	0.45	0.40	0.62	0.27	0.44	0.68	0.29
	2017	6704	0.58	0.76	0.47	0.42	0.65	0.28	0.45	0.71	0.28

*ANC* Antenatal care, *FBD* Facility based delivery, *SBA* Skilled birth attendance

**Fig. 1:**
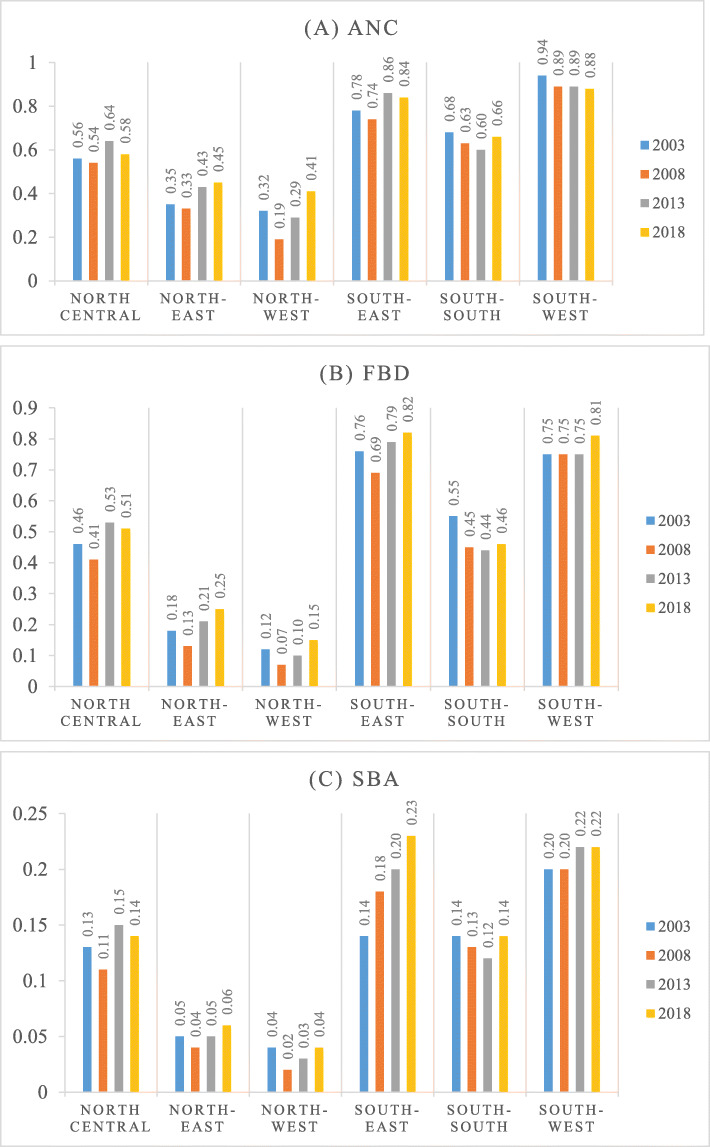
The proportion of antenatal care (ANC), facility-based delivery (FBD) and skilled-birth attendance (SBA) use across the six geopolitical zones of Nigeria 2003–2018

Figure 1a shows that all the southern geopolitical zones use ANC services more than their northern counterpart. Within the northern zone, the utilization of maternal care is lowest in the North-West zone. As shown in Fig. 1b, South-East and South-West zones use more FBD over the four survey years than the other geopolitical zones. As reported in Fig. 1c the South West, South-East, and North-Central zones have higher utilization of the SBA rate, while the North-West and North-East zones make less use of SBA.

**Fig. 2:**
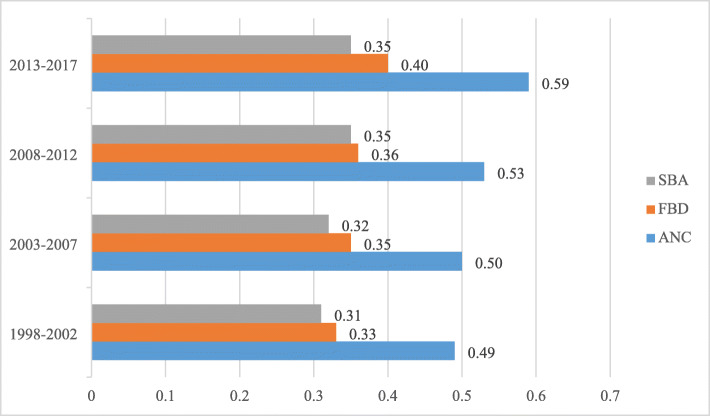
The proportion of antenatal (ANC), facility-based delivery (FBD), and Skilled-birth attendance (SBA) use across the survey period

Figure 2 shows the proportion of maternal healthcare use across the six geopolitical zones by four survey periods. The results indicate a pronounced increase in ANC use from 49 to 59 over the survey periods. However, this was not the case for SBA and FBD, which increased marginally from 31 to 35% and 33 to 40%, respectively, over the study period.

### Geographical inequalities in maternal healthcare utilization

Table 3 reports geographical inequalities in maternal healthcare use between rural and urban and across the geopolitical zones of Nigeria. The urban-rural rate ratios (RR) increased for ANC while it decreased for FBD and SBA over the study period. The relative advantage of urban women compared to rural women in ANC increased from 1998 (RR = 1.552) to 2017 (RR = 1.635). The relative inequality in FBD and SBA decreased from 1998 (RR = 2.980) to 2017 (RR = 2.371) and from 1998 (RR = 3.717) to 2017 (RR = 2.478), respectively. The magnitude of these changes was not statistically significant.

**Table 3:** Geographic inequalities in maternal healthcare use between the rural and urban area and across the six geopolitical zones of Nigeria: 2003–2018

Year	Inequalities between urban and rural areas						Inequalities across geopolitical zones					
	RR			RD			T			BGV		
	ANC	FBD	SBA	ANC	FBD	SBA	ANC	FBD	SBA	ANC	FBD	SBA
1998	1.552	2.980	3.717	0.271	0.385	0.218	0.356	1.053	3.041	0.024	0.158	0.240
1999	1.449	2.521	2.439	0.209	0.307	0.324	0.475	1.274	1.116	0.016	0.114	0.106
2000	1.902	2.433	2.003	0.368	0.296	0.298	0.489	0.907	0.815	0.052	0.069	0.062
2001	1.872	2.174	2.267	0.339	0.294	0.323	0.500	0.938	0.884	0.051	0.079	0.088
2002	1.952	2.060	2.155	0.352	0.269	0.308	0.558	1.006	0.902	0.057	0.068	0.077
2003	1.444	2.093	2.278	0.254	0.358	0.103	0.253	0.626	4.001	0.017	0.071	0.090
2004	1.740	2.602	2.545	0.333	0.381	0.388	0.384	0.941	0.879	0.039	0.123	0.117
2005	2.149	2.572	2.495	0.439	0.363	0.381	0.472	0.996	0.877	0.077	0.120	0.112
2006	2.058	2.613	2.539	0.393	0.375	0.395	0.535	0.961	0.840	0.067	0.124	0.117
2007	2.078	2.746	2.536	0.399	0.393	0.398	0.529	0.986	0.846	0.069	0.139	0.116
2008	2.104	2.751	3.286	0.431	0.408	0.293	0.379	0.795	1.681	0.070	0.131	0.185
2009	2.100	3.098	3.176	0.422	0.406	0.445	0.410	1.012	0.908	0.070	0.169	0.176
2010	2.021	2.790	2.970	0.382	0.384	0.431	0.487	0.944	0.864	0.064	0.139	0.156
2011	1.888	2.779	2.915	0.374	0.411	0.458	0.408	0.825	0.745	0.052	0.136	0.149
2012	1.979	2.679	2.825	0.380	0.392	0.437	0.462	0.859	0.783	0.060	0.128	0.142
2013	1.375	2.125	2.951	0.218	0.369	0.079	0.203	0.493	6.430	0.011	0.067	0.145
2014	1.627	2.468	2.521	0.317	0.359	0.394	0.267	0.815	0.723	0.029	0.105	0.109
2015	1.652	2.552	2.569	0.309	0.375	0.405	0.326	0.813	0.725	0.032	0.113	0.115
2016	1.676	2.334	2.351	0.302	0.357	0.391	0.391	0.736	0.639	0.034	0.092	0.094
2017	1.635	2.371	2.478	0.296	0.377	0.421	0.361	0.692	0.620	0.031	0.095	0.105
Time trend coefficients (*P*-value)	−0.002 (0.852)	0.000 (0.967)	0.006 (0.719)	0.001 (0.853)	**0.003 (0.024)**	0.007 (0.109)	**−0.007 (0.072)**	**−0.018 (0.004)**	−0.019 (0.743)	0.000 (0.836)	0.000 (0.883)	0.000 (0.927)

*RR* Rate Ratio, *RD* Rate Difference, *T* Theil Index, *BGV* Between Group Variance, *ANC* Antenatal care, *FBD* Facility based delivery, *SBA* Skilled birth attendance

The urban-rural rate differences (RD) indicate that women in urban areas use more maternal healthcare compared to their rural counterparts. In contrast to the RR results, the RDs show that absolute inequalities in maternal healthcare use between urban-rural areas increased for the whole study period. The increasing time trend coefficients of rate difference was significant for FBD. The estimated coefficient shows that the absolute gap in the utilization of FBD between urban and rural areas increased by 0.3% per year, over the period between 1998 and 2017 (Table 3). Both the T and BGV suggest that inequalities exist in maternal healthcare use across geopolitical zones in Nigeria. The T shows that relative inequalities in ANC, FBD across geopolitical zones declined by 7, and 1.8% per year, respectively. The BGV results do not suggest any changes in absolute inequalities in ANC, FBD, and SBA utilization across the geopolitical zones over time.

### Socio-economic inequalities in maternal care

Table 4 reports the relative and absolute education-related inequalities in maternal healthcare utilization among women of childbearing age for the survey period in Nigeria. The positive values of the RC and AC suggest a consistent concentration of all the three maternal healthcare services among well-educated women over the study period of 2003–2017. The extent of relative and absolute education-related inequalities in maternal healthcare utilization did not change over time.

**Table 4:** Education-related inequalities in maternal healthcare utilization among women aged 15–49 years in Nigeria: 2003–2018

Year	ANC		FBD		SBA	
	RC	AC	RC	AC	RC	AC
1998	0.508(0.298 to 0.717)	0.493(0.290 to 0.697)	0.674(0.561 to 0.786)	0.586(0.488 to 0.684)	0.542(0.418 to 0.665)	0.261(0.202 to 0.320)
1999	0.413(0.264 to 0.563)	0.412(0.263 to 0.562)	0.617(0.526 to 0.708)	0.497(0.424 to 0.570)	0.579(0.496 to 0.662)	0.493(0.423 to 0.564)
2000	0.578(0.492 to 0.664)	0.578(0.492 to 0.664)	0.663(0.590 to 0.736)	0.607(0.540 to 0.674)	0.646(0.569 to 0.723)	0.609(0.536 to 0.681)
2001	0.561(0.496 to 0.626)	0.561(0.496 to 0.626)	0.631(0.539 to 0.723)	0.571(0.488 to 0.654)	0.631(0.531 to 0.731)	0.581(0.489 to 0.673)
2002	0.529(0.469 to 0.589)	0.528(0.468 to 0.587)	0.617(0.545 to 0.688)	0.547(0.483 to 0.610)	0.600(0.532 to 0.667)	0.550(0.488 to 0.611)
2003	0.525(0.386 to 0.664)	0.469(0.345 to 0.593)	0.653(0.602 to 0.703)	0.644(0.595 to 0.694)	0.512(0.445 to 0.579)	0.202(0.176 to 0.229)
2004	0.565(0.501 to 0.629)	0.556(0.493 to 0.618)	0.660(0.624 to 0.695)	0.598(0.566 to 0.630)	0.669(0.635 to 0.703)	0.619(0.587 to 0.650)
2005	0.595(0.550 to 0.639)	0.594(0.550 to 0.638)	0.681(0.648 to 0.714)	0.606(0.577 to 0.636)	0.683(0.651 to 0.715)	0.632(0.602 to 0.662)
2006	0.577(0.543 to 0.612)	0.576(0.542 to 0.610)	0.660(0.624 to 0.696)	0.594(0.562 to 0.626)	0.664(0.630 to 0.698)	0.621(0.589 to 0.653)
2007	0.578(0.543 to 0.613	0.578(0.543 to 0.612)	0.671(0.636 to 0.707)	0.600(0.568 to 0.631)	0.666(0.633 to 0.699)	0.622(0.591 to 0.653)
2008	0.619(0.542 to 0.695)	0.607(0.532 to 0.682)	0.636(0.598 to 0.675)	0.603(0.567 to 0.640)	0.566(0.521 to 0.611)	0.400(0.368 to 0.432)
2009	0.650(0.599 to 0.701)	0.643(0.593 to 0.694)	0.676(0.641 to 0.711)	0.598(0.567 to 0.629)	0.694(0.659 to 0.729)	0.636(0.604 to 0.668)
2010	0.534(0.491 to 0.578)	0.534(0.491 to 0.578)	0.625(0.588 to 0.661)	0.566(0.533 to 0.598)	0.655(0.620 to 0.690)	0.608(0.576 to 0.641)
2011	0.555(0.517 to 0.592)	0.549(0.512 to 0.586)	0.626(0.591 to 0.660)	0.588(0.556 to 0.620)	0.657(0.623 to 0.691)	0.631(0.599 to 0.664)
2012	0.525(0.487 to 0.562)	0.524(0.486 to 0.561)	0.640(0.607 to 0.673)	0.595(0.565 to 0.626)	0.662(0.630 to 0.695)	0.630(0.599 to 0.661)
2013	0.542(0.360 to 0.724)	0.446(0.296 to 0.595)	0.614(0.549 to 0.678)	0.613(0.549 to 0.678)	0.487(0.418 to 0.555)	0.130(0.112 to 0.149)
2014	0.573(0.520 to 0.626)	0.521(0.472 to 0.569)	0.635(0.604 to 0.665)	0.598(0.569 to 0.627)	0.672(0.644 to 0.700)	0.650(0.623 to 0.676)
2015	0.525(0.482 to 0.568)	0.502(0.460 to 0.543)	0.650(0.621 to 0.680)	0.613(0.585 to 0.641)	0.681(0.653 to 0.709)	0.658(0.630 to 0.685)
2016	0.488(0.452 to 0.525)	0.481(0.445 to 0.517)	0.633(0.605 to 0.662)	0.610(0.583 to 0.637)	0.675(0.649 to 0.701)	0.665(0.639 to 0.691)
2017	0.496(0.464 to 0.527)	0.483(0.452 to 0.513)	0.608(0.576 to 0.640)	0.592(0.561 to 0.624)	0.658(0.629 to 0.687)	0.650(0.622 to 0.679)
Time trend coefficient (*P*-value)	0.0003(−0.004 to 0.005) (0.874)	−0.001(−0.006 to 0.004) (0.697)	−0.001(−0.003 to 0.0004) (0.132)	0.002(−0.0004 to 0.004) (0.104)	0.004(− 0.001 to 0.008) (0.132)	0.008(− 0.005 to 0.021) (0.198)

*RC*_*n*_ normalized relative concentration index, *AC*_*m*_ modified absolute concentration index, *ANC* Antenatal care, *FBD* Facility based delivery, *SBA* Skilled birth attendance

Table 5 reports the relative and absolute measure of wealth-related inequalities in maternal healthcare utilization in Nigeria. The positive values of the RC and AC indicate consistent pro-rich inequality in the utilization of ANC, FBD, and SBA in Nigeria over the survey period. Similar to the results of education-related inequalities, we did not find any change in the magnitude of wealth-related inequalities in maternal healthcare use in Nigeria.

**Table 5:** Wealth-related inequalities in maternal healthcare services among women aged 15–49 years in Nigeria: 2003–2018

Year	ANC		FBD		SBA	
	RC	AC	RC	AC	RC	AC
1998	0.560(0.329 to 0.790)	0.544(0.320 to 0.768)	0.636(0.507 to 0.765)	0.555(0.443 to 0.668)	0.540(0.417 to 0.664)	0.262(0.202 to 0.322)
1999	0.521(0.375 to 0.666)	0.519(0.375 to 0.664)	0.502(0.398 to 0.605)	0.406(0.323 to 0.490)	0.546(0.454 to 0.638)	0.467(0.388 to 0.545)
2000	0.510(0.416 to 0.605)	0.510(0.416 to 0.605)	0.623(0.534 to 0.712)	0.571(0.489 to 0.652)	0.638(0.549 to 0.726)	0.601(0.517 to 0.685)
2001	0.573(0.502 to 0.645)	0.573(0.502 to 0.645)	0.594(0.494 to 0.694)	0.539(0.448 to 0.630)	0.615(0.528 to 0.703)	0.568(0.487 to 0.649)
2002	0.509(0.434 to 0.585)	0.508(0.433 to 0.583)	0.522(0.422 to 0.621)	0.463(0.375 to 0.551)	0.559(0.463 to 0.654)	0.513(0.425 to 0.600)
2003	0.592(0.461 to 0.723)	0.529(0.412 to 0.646)	0.687(0.635 to 0.740)	0.679(0.627 to 0.731)	0.527(0.455 to 0.598)	0.208(0.180 to 0.236)
2004	0.641(0.582 to 0.700)	0.630(0.572 to 0.688)	0.682(0.642 to 0.722)	0.618(0.582 to 0.655)	0.687(0.647 to 0.726)	0.635(0.598 to 0.671)
2005	0.657(0.616 to 0.697)	0.656(0.616 to 0.697)	0.678(0.640 to 0.716)	0.603(0.569 to 0.637)	0.681(0.644 to 0.718)	0.630(0.596 to 0.664)
2006	0.627(0.593 to 0.662)	0.627(0.592 to 0.661)	0.660(0.620 to 0.700)	0.594(0.558 to 0.631)	0.680(0.642 to 0.718)	0.636(0.600 to 0.672)
2007	0.649(0.618 to 0.680)	0.648(0.618 to 0.679)	0.691(0.650 to 0.732)	0.617(0.581 to 0.654)	0.691(0.652 to 0.730)	0.646(0.609 to 0.682)
2008	0.716(0.655 to 0.777)	0.703(0.643 to 0.762)	0.667(0.625 to 0.709)	0.633(0.593 to 0.673)	0.606(0.555 to 0.657)	0.428(0.392 to 0.464)
2009	0.693(0.647 to 0.740)	0.686(0.641 to 0.732)	0.691(0.653 to 0.730)	0.612(0.578 to 0.647)	0.729(0.693 to 0.765)	0.668(0.635 to 0.701)
2010	0.633(0.594 to 0.672)	0.633(0.594 to 0.672)	0.668(0.629 to 0.707)	0.605(0.569 to 0.640)	0.700(0.661 to 0.738)	0.650(0.614 to 0.685)
2011	0.598(0.557 to 0.639)	0.592(0.551 to 0.632)	0.651(0.613 to 0.688)	0.611(0.576 to 0.647)	0.692(0.658 to 0.727)	0.666(0.633 to 0.698)
2012	0.605(0.571 to 0639)	0.604(0.570 to 0.638)	0.667(0.630 to 0.704)	0.621(0.586 to 0.655)	0.699(0.665 to 0.734)	0.665(0.633 to 0.698
2013	0.541(0.366 to 0.716)	0.445(0.301 to 0.588)	0.601(0.536 to 0.666)	0.601(0.536 to 0.666)	0.480(0.408 to 0.551)	0.128(0.109 to 0.148)
2014	0.598(0.544 to 0.651)	0.543(0.494 to 0.591)	0.606(0.569 to 0.643)	0.572(0.537 to 0.607)	0.659(0.626 to 0.692)	0.637(0.605 to 0.669)
2015	0.557(0.511 to 0.603)	0.532(0.489 to 0.576)	0.624(0.591 to 0.656)	0.588(0.558 to 0.619)	0.654(0.621 to 0.686)	0.632(0.600 to 0.663)
2016	0.483(0.443 to 0.524)	0.476(0.436 to 0.516)	0.596(0.564 to 0.629)	0.575(0.543 to 0.606)	0.647(0.618 to 0.675)	0.637(0.609 to 0.665)
2017	0.505(0.472 to 0.539)	0.492(0.459 to 0.525)	0.591(0.557 to 0.624)	0.575(0.543 to 0.608)	0.649(0.618 to 0.681)	0.642(0.611 to 0.673)
Time trend coefficient (*P*-value)	−0.0003(−0.006 to 0.005) (0.921)	−0.001(−0.008 to 0.005) (0.620)	0.001(− 0.003 to 0.006) (0.625)	0.004(− 0.0009 to 0.008) (0.107)	0.004(− 0.001 to 0.010) (0.129)	0.009(− 0.004 to 0.022) (0.181)

*RC*_*n*_ normalized relative concentration index, *AC*_*m*_ modified absolute concentration index, *ANC* Antenatal care, *FBD* Facility based delivery, *SBA* Skilled birth attendance

## Discussion

This study examined the geographical and socioeconomic inequalities in maternal healthcare services in Nigeria over the past twenty years. The results highlighted geographical inequalities in maternal healthcare services, especially for SBA and FBD across the six geopolitical zones in Nigeria. The results also suggest that women living in urban areas use more maternal healthcare compared to their rural counterparts. Essentially, the gap in the utilization of FBD between urban and rural areas increased/widened per year.

The results suggest inequalities in maternal care across the geopolitical zones in Nigeria. The finding highlights the perennial entrenchment of North-South differences despite maternal healthcare interventions [37] The intermittent geographic inequalities in the SBA and FBD could be because of the perennial poor socioeconomic development of the northern part of Nigeria [10, 19] which may result in lower utilization of maternal care in northern zones.

Results also indicate consistent socioeconomic inequalities in ANC, FBD, and SBA. Both relative and absolute measure of inequalities indicated higher concentration of maternal healthcare services among the better-off and well-educated women over the four survey years despite the concerted efforts of government interventions such as the introduction of free maternal and child health [38] to contain the abysmal maternal mortality ratio in the country.

The later findings are similar to earlier studies that show pro-rich inequalities in maternal healthcare utilization in Ghana [39] and Nigeria [40]. These results provide important evidence that may assist the health stakeholders to redouble their efforts toward achieving the Sustainable Development Goals (SDGs) three targets of reducing the global maternal mortality ratio to less than 70 per 100,000 live births by 2030 [41]. With the government Free Maternal and Child Health Program aimed at decreasing the high maternal mortality by increasing access to maternal health services, evidence indicates that such intervention leads to an increased percentage of access to SBA thereby reducing maternal mortality [38, 42].

Further, findings show that the northern geopolitical zones especially the North-West zone compared with their southern counterparts lag in the utilization of maternal healthcare services. This is not surprising because of the wide gap in socio-economic development between the northern and southern parts of the country [10]. Studies by Obiyan and Kumar [5] and Nghargbu and Olaniyan [40] also emphasized that wealth status and education were the major factors driving inequality in maternal healthcare utilization in Nigeria. Nghargbu and Olaniyan [40], shows that SES rather than the need for healthcare mainly determine demand for maternal healthcare.

The pronounced inequalities in maternal healthcare services in the northern geopolitical zone are exacerbated by several supply-side factors (lack of accessibility, availability, quality, and comprehensiveness of health services) and demand-side factors (social, economic, and cultural) as confirmed by Obiyan and Kumar [5]. As healthcare costs, transportation, and quality of services were identified as barriers for women seeking maternal health services in Nigeria [37], addressing supply-side barriers alongside demand-side factors may lead to an improvement in the maternal care use in Nigeria, especially among low SES women [6].

To address inequalities in maternal care in Nigeria, the political will of both sub-national and national governments is needed for context-specific interventions. National health systems are key in addressing health inequalities and no state or geopolitical zone should face levels of health inequalities that are avoidable [43]. The northern geopolitical zone should give special attention to the upgrade of hospitals for the uptake of obstetric care [44, 45] so that during an emergency, pregnant women should have access to an appropriately equipped health service. As distance is an important barrier to seeking healthcare, especially in rural areas [46], obstetric care must be located within reasonable reach of the people who should benefit from it [46, 47].

This study shows a positive education gradient in the utilization of maternal healthcare services. The education level of women has been found to affect their use of healthcare facilities in other studies [45]. Thaddeus and Maine [48] also found a significant positive association between the use of prenatal care services and the level of women’s education. This is important, especially for the North-West and North-East geo-political zones where the female literacy rate is as low as 38% [19], which calls for action to address the trend and increase maternal healthcare services uptake.

Our descriptive results indicate that women of the Christian religion utilize more key maternal healthcare services compare with their Muslim counterparts. The higher use of maternal healthcare services by the Christian women in the South could be due to their higher level of education compared with the Muslim women in the North [49]. This may explain the lower utilization of the maternal healthcare services in the North-East and North-West geopolitical zones where Islam is the main religion. Evidence shows that most husbands practicing Islam discourage their wives from going out without their permission [50]. This presents a barrier to use maternal healthcare for Muslim women, especially when the husband is away from home [48].

One of the strengths of this paper is that the study used nationally representative data that allows the generalization of findings to the entire country. The use of several measures of inequality to assess geographical and socioeconomic inequalities in maternal healthcare is another strength of the study. This study, however, is subject to some limitations. First, the self-reported maternal healthcare use in DHS may be subject to recall bias. Second, although information on maternal healthcare utilization is obtained from pregnancy and delivery occurred between two to four years before the survey year, the WI as one of SES indicators is constructed from information collected for the survey year. As changes in household wealth occur in the long-run, we considered the WI for the survey year to be a reasonable proxy for recent years.

## Conclusion

Geographical and socioeconomic inequalities in maternal healthcare utilization prevail in Nigeria. Specifically, the results of this study demonstrated that the utilization of maternal healthcare is lower among poorer and less-educated women, as well as those living in rural areas and North-West and North-East geopolitical zones. Thus, priority focus should be on implementing strategies that increase the uptake of maternal healthcare services among these groups in Nigeria.

## Data Availability

Data for this study is publicly accessible at the DHS website: https://www.dhsprogram.com/data/available-datasets.cfm

## Abbreviations

ANC: Antenatal Care
DHS: Demographic Health Survey
FBD: Facility-based delivery
SBA: Skilled birth attendance
RR: Rate ratio
RD: Rate difference
RC: Relative concentration index
AC: Absolute concentration index
T: Theil index
BGV: Between-group variance
SES: Socioeconomic status
PCA: Principal component analysis
SSA: Sub-Saharan Africa
WHO: World Health Organization
